# Autoimmune gastritis in a male adolescent with cerebellar involvement: A case report

**DOI:** 10.1016/j.amsu.2022.104228

**Published:** 2022-08-06

**Authors:** Prakash Sapkota, Sadikshya Bhandari, Bhuwan Thapa, K.C. Sajana, Pramita Shrestha

**Affiliations:** a Department of Internal Medicine, Dhulikhel Hospital, Kathmandu University Hospital, Kathmandu University School of Medical Sciences, Dhulikhel, Kavre, Nepal; b Department of Public Health and Community Programs, Dhulikhel Hospital, Kathmandu University Hospital, Kathmandu University School of Medical Sciences, Dhulikhel, Kavre, Nepal

**Keywords:** Adolescent, Autoimmune gastritis, Cerebellar, Neurological manifestation, Non-vegetarian

## Abstract

**Introduction:**

Autoimmune gastritis is an immune mediated disorder characterized as anti-intrinsic factor and anti-parietal cell autoantibodies directed against intrinsic factor and parietal cells of the stomach respectively, leading to vitamin B12 deficiency. When the disease remains undiagnosed and untreated, it may lead to neurological complications and even fatal anemia.

**Case study:**

We exemplify a non-vegetarian male adolescent case with the neurological symptoms such as bilateral leg weakness, unsteady gait, slurred speech, vertigo, slowed movement, lethargy, and impaired joint sensation. None of his family members had such illness. His hemoglobin was normal with serum vitamin B12 level 105 pg/mL and anti-intrinsic factor antibody titer positive. A presumed diagnosis of cobalamin deficiency with involvement of the cerebellum, dorsal column and peripheral nerves was made. His symptoms recovered gradually and later completely (after 6 months) after the intramuscular vitamin B12 therapy.

**Clinical discussion:**

The indexed rare adolescent case had auto immune gastritis showing neurological manifestation with more pronounced cerebellar features and vitamin B12 deficiency under the non-vegetarian diet consumption. Previous studies had reflected auto immune among adolescents but contrasted some of the clinical features.

**Conclusion:**

For the prompt and precise diagnosis of the autoimmune gastritis and to prevent further complications, some of the rare conditions such as deficiency with a non-vegetarian diet, neurological manifestation including cerebellar involvement without anemia should also be considered along with other relevant symptoms. The heightened awareness for timely surveillance and treatment will contribute in reduction of such unusual cases.

## Highlights

• A rare adolescent with autoimmune gastritis and vitamin B 12 deficiency
• The case involved cerebellum with other neurological manifestation without anemia.
• Timely and the uninterrupted intramuscular vitamin B12 therapy improved the clinical and radiological findings.

## 1 Introduction

Autoimmune gastritis is an immune mediated disorder in which autoantibodies are directed against both intrinsic factor and parietal cells, affecting the fundus and body of the stomach, where parietal cells are replaced by atrophic glands, which may undergo metaplastic change [1]. Both congenital and juvenile forms of autoimmune gastritis are thought to follow an autosomal recessive inheritance pattern [2]. The clinical presentation of autoimmune gastritis is often insidious and symptoms vary within the course of illness. Symptoms may include fatigue, pallor, paresthesia, incontinence, psychosis, and generalized weakness. When the disease remains undiagnosed and untreated for an extended period, it may lead to neurological complications and even fatal anemia [2]. Limited studies has depicted a rare adolescent non vegetarian case of autoimmune gastritis with vitamin B12 deficiency, involving cerebellum with other neurological manifestation without anemia. The case's clinical and other characteristics, presented in this study will generate insightful information regarding such rare conditions in the existing literatures.This study aims to present an adolescent case with manifestation other than the usual autoimmune gastritis.

## 2 Case report

A 17 years old male presented with complaints of insidious onset of both leg weakness for about two and half months noticed when he had trouble holding onto his slippers with his feet. In a span of a month, his intensified weakness resulted in difficulty in walking without support. About a month after the onset of his weakness, he had an undocumented bout of fever which was associated with non-productive cough. This led to raised weakness followed by imbalanced walking, intermittent tremor-like movement in his legs and arms.

Since the onset of his symptoms, he had excessive sweating, severe lethargy, easy fatigue, and decreased touch and warm/cold sensation in lower limb. In the last two months, he had trouble coming up with the desired words and was easily distracted. He forgot the names of his family, friends and had difficulty in solving regular mathematics. He also complained of unsteady gait, slurred speech and vertigo. He also reported swinging movement of his trunk while standing and walking, even with support. However, he denied back pain; stool, urinary incontinence, urinary retention, constipation; dizziness, double vision, decreased vision, and decreased taste sensation, deviation of angle mouth, vomiting and headache. Prior to the onset of the symptoms, he did not have history of diarrhea, shortness of breath, sore throat, blurring of vision and photophobia. He had no significant family history, past history of tuberculosis, diabetes mellitus or other significant comorbidities. He denied history of trauma, illicit drug use, use of herbal remedies, unprotected sexual intercourse, or insect bite. His diet was non-vegetarian and consumed both red and white meat.

His vitals were stable and was well oriented to time, place an person with Glasgow coma scale scored 15 and mini mental state examination (MMSE) of 20/30 with problem in recall, attention and calculation. General physical examination was normal without associated pallor, glossitis, stomatitis and lymphadenopathy. Pupils were equal, round and reactive; and normal fundus without any feature of optic atrophy on fundoscopy. The cranial nerve examination was normal. In the motor examination, bulk, power and tone was normal in all limbs. Power in the ankle flexors and extensors was 2/5 bilaterally, 3/5 on knee flexors and extensors and 4+/5 on hip flexors and extensors, whereas power was normal in upper limb. Deep tendon reflex (DTR) in both ankle and knee was absent whereas it was decreased in both biceps tendon and supinators. He also had an intentional tremor and dysmetria with staggering gait but nystagmus and dysdiadochokinesia was absent. His finger nose test and heel shin test was normal. Bilateral Babinski sign was present along with decrease joint position, and absent vibration sense over both upper and lower limbs bilaterally. Temperature and fine touch sensation decreased in both lower limb but was intact in upper limb and upper boarder of loss of this sensation could not be delineated. The abdominal, cardiovascular and respiratory examination were normal. With these findings a differential diagnosis of Vitamin B12 deficiency, demyelinating disease like multiple sclerosis (MS), viral or autoimmine encephalomyelopathies was formed and investigations were sent.

On investigations, as seen in Table 1, complete blood count was normal. Normocytic normochromic red blood cells with mean corpuscular volume (MCV) of 100 fl and hyper segmented neutrophils were seen in peripheral smear. He had an elevated erythrocyte sedimentation rate (ESR) i.e. 28 mm/hr and normallipid profile and cerebro spinal fluid (CSF). With the normal levels of protein and inflammatory cells in the CSF, we were able to rule of encephalomyelopathies secondary to viral infections and autoimmune causes. His serum vitamin B12 level was 105 pg/ml (normal range: 178.0–883.0) and folic acid leveled 7.1ng/ml (normal range: >3.0) and vitamin E level was normal (7.41mg/dl). His stool examination was normal without any ova or trophozoites of parasites and stool occult blood test was negative.

**Table 1:** Laboratory investigation findings of the indexed case.

Parameters	Findings (admission)	Findings (after 6 months)	Normal value
Complete blood counts			
Neutrophils (%)	66	76	45–75
Lymphocytes (%)	24	18	20–45
Total leukocyte count (x10 3/μL)	10.8	–	4.0–11.0
Platelets (x10 3/uL	272	271	150–450
Mean corpuscular volume (MCV) (fl)	100	73	80–100
Erythrocyte sedimentation rate (ESR) (mm/hr)	28	–	0–15
Peripheral blood smear (PBS)	Normocytic normochromic	Normocytic normochromic	
Hemoglobin (g/dl)	13.8	14.1	13–17
**Cerebro spinal fluid (CSF)**			
Protein (mg/dL)	50.0	–	15–45
Adenosine deaminase (ADA) (U/L)	4	–	<10
Sugar (mg/dL)	52	–	40–80
Cell	Nil	–	
Acid fast bacilli	Not seen	–	
Culture	No organism growth	–	
Gram stain	No microorganism seen	–	
**Other biochemical parameters**			
Vitamin B12 (pg/mL)	105	845	178.0–883.0
Folic acid (ng/mL)	7.1	–	>3.0
Vitamin E (mg/L)	7.41	–	6.00–10.00
Parietal cell antibody	Positive	–	
Titer	1:40	–	
Thyroid stimulating hormone (TSH) (μIU/mL)	4.62	–	0.35–4.94
**Serology**			
HIV	Negative	Negative	

**Fig. 1:**
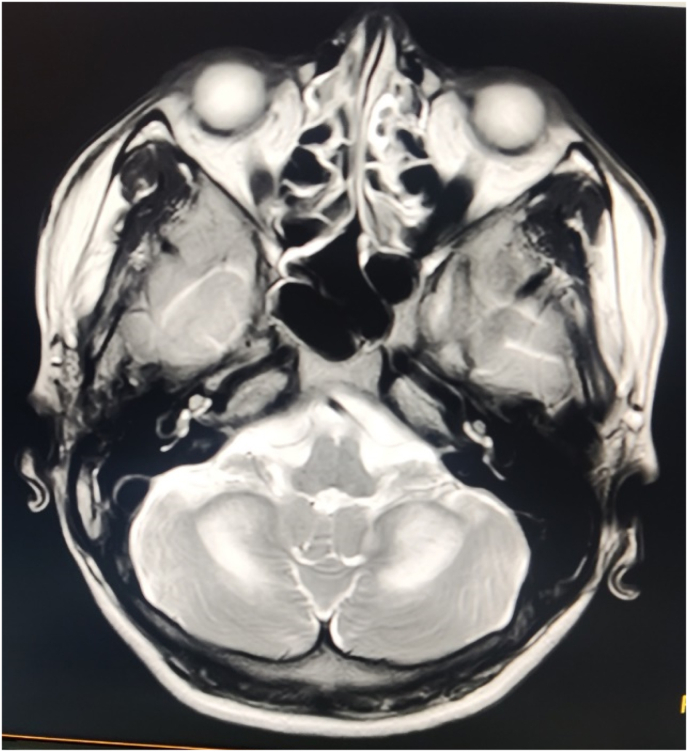
MRI scan of cerebellum showing hyper intensity along the medial aspect of the bilateral cerebellar hemisphere.

The ultrasound examination of the abdomen and pelvis showed normal spleen with focal fatty sparing in the perihilar region in the liver. A magnetic resonance imaging (MRI) of brain (Fig. 1) showed symmetrical T2 hyper intensity along the medial aspect of the bilateral cerebellar hemisphere presumed to be demyelination. The spine MRI showed normal report but along with mild sub-acute combined degeneration of spinal cord, the cerebral features was more pronounced.

A presumed diagnosis of ataxia secondary to cobalamin deficiency with involvement of the cerebellum, dorsal column and peripheral nerves was made following consultations with a neurologist. The peripheral nerve involvement were determined following the clinical features of the indexed case, while the nerve conduction study was not done for this case. Treatment was started with intramuscular (I.M.) methylcobalamine 1000 mcg on alternate days for 5 doses. With treatment vibration, position sense, touch and temperature sensations improved along with cognitive symptoms. He was discharged on 10th day with Inj. Methylcobalamine 1000 mcg I.M. weekly injection and he was advised to follow up at the outpatient clinic after 1 week with remaining investigation reports. At the time of discharge, he was able to stand and walk few steps without support and had improved ataxia. Although a clinically isolated syndrome of multiple sclerosis could be suspected even in the presence of a superimposed vitamin deficiency, but was less likely as symptoms in multiple sclerosis develop over hours to days gradually remit over weeks to months, although not completely unlike that seen in this patient. Furthermore, with treatment of Vitamin B12 deficiency the patient showed rapid and marked improvement as such this possibility was discarded and specific investigations for multiple sclerosis were not done.

Most of the aforementioned cognitive cerebellar and motor symptoms, gait, joint position, vibration sensation, DTR were steadily improved. He had normal plantar reflex, his intentional tremor subsided and was able to walk without support on subsequent follow up.

Anti-parietal cell antibody titer (1:40) was positive and diagnosed with ataxia secondary to vitamin B12 deficiency due to autoimmune gastritis without anemia. He was treated with intramuscular Vitamin B12 weekly till the symptoms subsidized completely, and later reduced to a monthly dose which will be continued every three months throughout his life. However, he refused to perform an upper gastrointestinal endoscopy investigation.

**Fig. 2:**
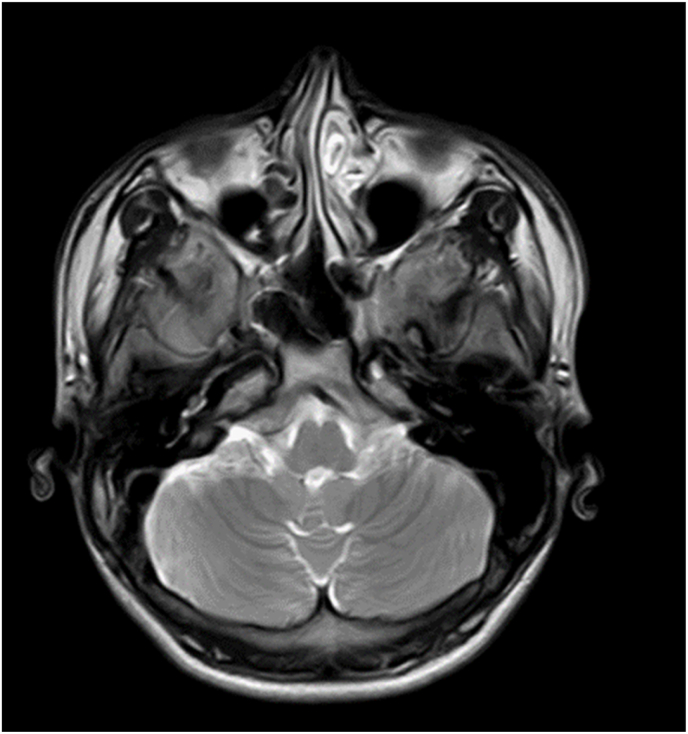
MRI follow up scan of cerebellum showing no altered signal intensity along the medial aspect of the bilateral cerebellar hemisphere.

During the follow up after 3 months, his cognition had improved with 28/30 MMSE score and he had normal vitamin B12 level. In the subsequent follow up in 6 months, his MRI scan (Fig. 2) showed no altered signal intensity along the medial aspect of the bilateral cerebellar hemisphere. The reinvestigated laboratory parameters were within the normal range. This case study adopted CARE items following CARE checklist and guideline [3].

## 3 Discussion

Autoimmune gastritis is the most common cause of cobalamin deficiency throughout the world [4]. The prevalence is hugely affected by age, ranging from 0.1% in the general population to 1.9% in the elderly population above the age of 60 years [5]. It is thus considered a condition of older individuals but some other study also has stated the onset of inflammation may occur in adolescents, preceding the physical and biochemical findings of iron deficiency anemia or B12 deficiency [6]. This study also exemplified that adolescent cases may present with rare clinical manifestations of cobalamin deficiency.

Also, the indexed case can be considered rare as the patient was presentedwith neurological manifestation without anemia which is seldom reported in other studies. A similar case was seen in Japan when a 45-year-old woman presented worsening mild weakness, numbness in bilateral lower limbs, and gait disturbance who had macrocytosis without anemia and mild reduction in total serum vitamin B12 level; who after administration of vitamin B12 had improvement in her neurological symptoms as well as macrocytosis [7]. However, such cases are often even rarer during adolescence in whom cobalamin deficiency most often occurs as a result of dietary deficiency commonly due to veganism [8]. In the indexed case, although he consumed both red and white meat, he still had the vitamin B12 deficiency, signifying the dearth of association between his dietary habits and cobalamin deficiency.

The diagnosis of autoimmune gastritis remains complicated in such circumstances as it has diverse clinical manifestations and with spuriously normal or high cobalamin levels, normocytic or microcytic anemia, and neurologic manifestations without anemia or macrocytosis [9]. Diagnosis of the cobalamin deficiency often requires the level of serum cobalamin less than 200 pg/mL (ng/L) using automated enzymatic chemiluminescence assays as seen in the exemplified case. Patients with serum cobalamin levels between 200 and 400 ng/L may have true deficiency and are often considered borderline [5]. The case reported in this study had inadequate cobalamin level, lesser than other studies. While the findings of the peripheral blood smear were similar to other studies. His mean corpuscular volume (MCV) was in the upper normal range. Other vitamins including Vitamin E may play a role in the development of neurological abnormalities as it is also necessary for the maintenance of integrity and function of nerves and its deficiency results in a similar presentation as of Vitamin B12 deficiency in the absence of anemia [10]. However it was seen to be normal in the indexed case thus supplementation was not needed. Iron deficiency is a common presenting feature of achlorhydria secondary to autoimmune gastritis, more commonly seen in adolescents and in females. Thus needs to be monitored if anemia is suspected in the patient [11]. However, despite the neurological symptoms the indexed case did not have symptoms of anemia and with MCV in upper limit (100fl), such a detailed investigation to determine iron levels was also not conducted. We did not investigate other vitamins and minerals including calcium or even Vitamin D as there were no signs or symptoms in the patient which could be explained by their deficiencies.

Antiparietal cell antibody testing is relatively sensitive in 85% of Autoimmune gastritis but are not specific as they may also be present in patients with autoimmune polyendocrine diseases and 3–10% of normal healthy populations [5]. This study presented a case with positive parietal cell antibody with 1:40 titer.

Autoimmune disease commonly present together with hypothyroidism, vitiligo and diabetes [12]. However, the case in this study did not have any history of other autoimmune diseases. The spinal cord involvement with MRI finding of a symmetrical abnormally increased T2 signal intensity, commonly confined to posterior or posterior and lateral columns in the cervical and thoracic spinal cord is specific for sub-acute combined degeneration of spinal cord secondary to cobalamin deficiency [13]. In contrast, the case in this study had no spinal cord involvement as seen in his MRI report. Brain involvement has been reported in B12 deficiency with fluid attenuated inversion recovery and T2-weighted images demonstrating extensive areas of a high-intensity signal in the periventricular white matter [13]. The MRI findings of the indexed case illustrated the involvement of demyelination of the cerebellar part of the brain and the cerebellum hyper intensity along the medial aspect of the bilateral cerebellar hemisphere which is less common and a rare condition for the vitamin B12 deficiency. However, in a follow up MRI (after 6 months), the cerebellum showed no altered signal intensity.

## 4 Conclusion

Although the inflammatory process may start earlier in life, autoimmune gastritis still remains a rare condition in adolescents. For the prompt and precise diagnosis and to prevent further complications, some of the rare conditions such as deficiency with a non-vegetarian diet, neurological manifestation including cerebellar involvement without anemia should also be considered along with other relevant symptoms. The timely and the uninterrupted intramuscular vitamin B12 therapy intruded the autoimmune gastritis in a non-vegetarian adolescent. However, given the potential for metaplastic transformation and dysplasia, the heightened awareness of its rare presentation is required for surveillance and treatment wherever indicated.

## Ethical approval

Not applicable as this is a case report.

## Sources of funding

We also state that there was no source of funding for this study.

## Author statement

Prakash Sapkota: Manuscript writing and editing, guarantor.

Sadikshya Bhandari: Manuscript writing and editing.

Bhuwan Thapa: Manuscript writing and editing.

Sajana KC: Manuscript writing and editing.

Pramita Shrestha: Manuscript editing.

## Consent

Written informed consent was obtained from the patient for publication of this case report and accompanying images. A copy of the written consent is available for review by the Editor-in-Chief of this journal on request.

## Registration of research studies

Name of the registry: N/A.

Unique Identifying number or registration ID:

Hyperlink to your specific registration (must be publicly accessible and will be checked):

## Guarantor

Dr. Prakash Sapkota; Resident, Department of Internal Medicine, Kathmandu University School of Medical Sciences, Nepal.

## Provenance and peer review

Not commissioned, externally peer reviewed.

## Declaration of competing interest

We declare that there are no conflicts of interest amongst the authors.
